# Flexible delivery of opioid agonist treatment during COVID-19 in Norway: qualitative and quantitative findings from an online survey of provider experiences

**DOI:** 10.1186/s12913-023-09959-7

**Published:** 2023-09-07

**Authors:** Rebecca McDonald, Anne Berit Bech, Thomas Clausen

**Affiliations:** 1 Norwegian Centre for Addiction Research (SERAF), Institute of Clinical Medicine, Oslo University, P.O. Box 1171, Blindern, Oslo, 0318 Norway; 2 Faculty of Social and Health Sciences, Inland University of Applied Sciences, P.O. Box 400 Vestad, Elverum, 2418 Norway

**Keywords:** Opioid substitution treatment, Opioid use disorder, Buprenorphine, Methadone, Telemedicine

## Abstract

**Background:**

For patients receiving daily opioid agonist treatment (OAT) for opioid dependence, several countries relaxed treatment guidelines at the beginning of the COVID-19 pandemic. This involved longer take-home intervals for methadone and buprenorphine doses as well as a reduction in supervised dosing and drug screening. To date, little is known about the medium or long-term experience of OAT deregulation. Therefore, we conducted a survey to explore how OAT providers perceived greater flexibility in OAT service delivery at the end of the second year of the pandemic.

**Methods:**

Nationwide cross-sectional study of twenty-three OAT units in 19 publicly funded hospital trusts in Norway. OAT units were sent a 29-item online questionnaire comprising closed-format and open-ended questions on treatment provider experiences and changes in OAT service delivery during the past 12 months (January to December 2021).

**Results:**

Twenty-three (of whom female: 14; 60.8%) managers or lead physicians of OAT units completed the questionnaire reporting that, in 2021, most OAT units (91.3%,* n* = 21) still practiced some adjusted approaches as established in the beginning of the pandemic. The most common adaptions were special protocols for COVID-19 cases (95.7%,* n* = 22), increased use of telephone- (91.3%, *n* = 21) and video consultations (87.0%,* n* = 20), and longer take-home intervals for OAT medications (52.2%, *n* = 12). The use of depot buprenorphine also increased substantially during the pandemic. According to the OAT providers, most patients handled flexible treatment provision well. In individual cases, patients’ substance use was identified as key factor necessitating a reintroduction of supervised dosing and drug screening. Collaboration with general practitioners and municipal health and social services was generally perceived as crucial for successful treatment delivery.

**Conclusions:**

Overall, the Norwegian OAT system proved resilient in the second year of the COVID-19 pandemic, as its healthcare workforce embraced innovation in technology (telemedicine) and drug development (depot buprenorphine). According to our nationally representative sample of OAT providers, most patients were compliant with longer take-home doses of methadone and buprenorphine. Our findings suggest that telemedicine can be useful as adjunct to face-to-face treatment and provide greater flexibility for patients.

## Background

In response to the introduction of COVID-19 social distancing measures in March 2020, drug treatment services had to quickly adapt their operating procedures to ensure continuity of care while mitigating risk of viral infection. This included opioid agonist treatment (OAT) units which provide daily buprenorphine or methadone to patients with opioid use disorder (OUD), for whom access to and retention in OAT is associated with reduced mortality [1]. Supervised dosing, i.e., the oral administration of OAT medication to the patient under supervision of a treatment staff member, is a common measure in OAT to prevent diversion of the medication [2]. For patients who remain in OAT, access to take‐home and/or unsupervised doses can be used as positive reinforcement following initial stabilization.

At the onset of the pandemic, OAT guidelines were temporarily relaxed in several countries [3–6], resulting in decreased requirements for in-person visits or supervised dosing, less drug screening, more OAT take-home doses as well as the introduction of telemedicine (i.e., remote appointments between a patient and their healthcare practitioner using phone or video). International studies show that such greater flexibility in the OAT system during the first year of the pandemic was largely perceived as positive, both by treatment providers and patients [7–11]. For example, in the United States (US), telemedicine allowed providers to initiate patients onto buprenorphine without an in-person appointment during the pandemic [12]. Providers reported that this policy change improved treatment access in urban and rural settings, also among vulnerable populations (homeless individuals; people leaving incarceration; people attending needle and syringe programs), by removing common barriers such as stigma and lack of transport [13, 14].

In Norway, a COVID-19 survey [15] conducted in July–August 2020 confirmed that more than three quarter (76–95%) of OAT units nationwide had implemented telemedicine, extended the OAT medication take-home intervals beyond seven days, and reduced supervised dosing and drug screening [15]. However, data collection in this and other studies was limited to the first wave of the pandemic in 2020, when society was fully immersed in lockdown [7–11, 16]. Meanwhile, comparatively little is known about the medium or long-term experience of COVID-19-related adaptions, including how providers have perceived flexibility in treatment as society reopened into a “new normal”.

To address this gap in knowledge, we conducted an OAT provider survey in late 2021, i.e., at the end of the second year of the pandemic. Such research can inform addiction service delivery beyond the end of the pandemic as well as during ongoing OAT reform discussions. One important aspect of a resilient health system is its capacity to adapt and learn from experiences in times of stress and shock [17]. Thus, our research question was: How did OAT providers in Norway experience adaptions in service delivery, including the use of telemedicine, in the second year of the COVID-19 pandemic?

## Methods

### Study design

This nationwide descriptive study is based on a cross-sectional online survey of the leaders (i.e., manager or lead physician) of OAT units across 19 publicly funded hospital trusts in Norway. The study was conducted in accordance with the STROBE statement [18].

### Setting

Specialized OAT units across all publicly funded hospital trusts in Norway are responsible for the initiation and delivery of OAT treatment. The OAT units operate without waiting lists, in collaboration with general practitioners (GPs) and municipal health and social services [19]. OAT induction is initiated by the OAT units on an in- or outpatient basis, and prescribing can later be transferred to primary care services. OAT is provided free of charge to patients and as life-long treatment for many.

As of 2021, the Norwegian OAT population has encompassed 8,198 patients, relative to approximately 10,500 high-risk opioid users (i.e., opioid use by injection or long duration/regular use), among a total population of 5.5 million [20, 21]. Roughly one third of Norwegian OAT patients are women. At an average age of 47 years [21], Norwegian OAT patients are among the oldest in Europe.

In 2021, buprenorphine was the most prescribed OAT medication in Norway, accounting for two thirds of the OAT population in its three formulations: Buprenorphine monotherapy (38%), buprenorphine/naloxone (8%), and – following its introduction onto the Norwegian market in 2019 – buprenorphine depot injection (15%). A third of the OAT population (33%) received methadone treatment, for which prescribing trends have declined over the past decade. Thirty-five percent of patients were prescribed their OAT medication by their GP [21].

### Participants

Recruitment occurred in December 2021, coinciding with the biannual OAT leader meeting in Norway, and ended on 21 January 2022. At this meeting, 1–2 representatives from each OAT unit convene to discuss recent developments in clinical practice. The mailing list for the OAT leader meeting, which comprised 42 recipients representing all 19 hospital trusts responsible for OAT delivery in Norway, was used for distribution of the online questionnaire, and follow-up reminders were sent by email. We aimed to receive a response from at least one OAT unit representative from each of the 19 hospital trusts, with the possibility of multiple OAT units per hospital trust responding.

Respondents were eligible to participate in the survey if they were either the manager or the lead physician of the OAT unit. We specifically requested to receive only one completed online questionnaire per OAT unit. If survey recipients worked in the same OAT unit, they were given the option to discuss the survey items and submit one single joint response. Respondents were asked to indicate their gender as well as the number of patients treated at their OAT unit. Respondents were not reimbursed for their participation.

The study was conducted as part of a service evaluation for the Norwegian Directorate of Health and did not require ethical review. Only program-level data without personally identifiable patient data were collected. The study was approved by the Data Protection Authority (registration number: 552107) at the University of Oslo, and all data were stored in compliance with university standards. No external funding was received.

### Measures

The online questionnaire was a modified and extended version of the COVID-19 survey from July–August 2020 [15] and covered questions related to treatment provider experiences and changes in OAT service delivery during the past 12 months (January to December 2021), i.e., the second year of the COVID-19 pandemic. The questionnaire contained 29 questions in total. Among these, 25 questions were in closed format (categorical) which assessed the duration of take-home doses, frequency of supervised dosing, frequency of drug screens, COVID-19 vaccination among patients, and the use of telemedicine. The remaining four questions were in open-ended format, enquiring: positive and negative experiences in past 12-month OAT provision, changes in provider collaboration, and permanent changes to clinical practice. In addition, free-text comments were possible on 12 of the 25 closed-format questions.

### Analysis

#### Quantitative data

Quantitative data were described as frequencies and percentages if categorical, or medians and minimum and maximum values if continuous (non-normally distributed). IBM SPSS Statistics Version 26 (Chicago, IL) was used for descriptive statistical analysis.

#### Qualitative data

Respondents’ written qualitative responses to open-ended questions were analysed following the basic principles of Iterative Categorization (IC) (for fuller description, see Neale et al. [22, 23]). This method was deemed appropriate as it has been previously applied to the analysis of qualitative text comments from another COVID-19 online survey, where no qualitative interviews were conducted either [24]. The stages in IC include transcription, familiarization, anonymization, logging, coding, analyses preparation, descriptive analyses, and interpretative analyses [23]. The open-text qualitative responses were listed verbatim in a Word document. The qualitative analysis was led by the joint first two authors. Both RM and ABB read and re-read the content. Each transcription was anonymized using a unique identifier. Coding and analyses involved reviewing all text segments line-by-line, converting the text segments into simple statements where appropriate, then iteratively grouping these statements into meaningful categories based on deductive codes derived from the questionnaire. Deductive codes were supplemented by inductive codes emerging from the data; however, these were not relevant to the aims of this study and therefore not included. Important themes and categories identified during descriptive analyses were further organized by two main categories (with in total six sub-categories): (1) Experiences of changes in service delivery, and (2) Experiences of changes in collaboration. Responses relating to either of the two categories headings were further organised into six sub-categories as reported below (see also Table 1). Interpretative analyses also included differentiation (checking data for similarities and differences), and externalizing (i.e., discussion). We provide free text extracts to highlight key points relating to the subcategories.

**Table 1:** Main categories, sub-categories (in italics), and select corresponding quotes from qualitative analysis

	**Quotes**
**Changes in service delivery**	
* Medication dispensing*	“Positive collaboration with most patients in terms of infection control. Patients spend less time collecting the medication, which improve their quality of life. Home delivery of the medication has allowed us to get a better sense of the patients’ living situation and needs” (ID #1) “It is easier to relax safety measures than to tighten these again” (ID #5) “We have moved form a standardized approach to more individual treatment in each case. Patients express more satisfaction with OAT in every way. Treatment availability is improved with telemedicine and more outreach work. We have increased our availability across all channels. lt is easier for our patients to contact us. We have seen surprisingly good effect of the reduction in supervised dosing and drug screens” (ID #16)
* Depot buprenorphine*	“Many patients express positive experiences and satisfaction with [depot buprenorphine], where for some the pandemic has been decisive for this medication choice.” (ID #04)
* Drug screening*	“urine drug screening has been significantly reduced” (ID #23) “[we] largely switched to saliva samples” (ID #01, ID #10) “During the entire COVID-19 period, individual assessments have been made [for] urine drug screening, supervised dosing, pick-up intervals for medications, with the aim to strike a balance between treatment safety and risk of infection” (ID #08) “Recently, urine drug screening has resumed for some patients where there has been a need to assess their drug use” (ID #11)
* Telemedicine*	“phone and video cannot compensate for face-to-face contact” (ID #19) “[makes us] lose the interaction of joint meetings with the patient” (ID #05) “[makes us lose] essential observations in treatment, such as a patient’s smell, skin color, tremor, etc.” (ID #01) “[some patients] have become more isolated as there are fewer face-to-face meetings” (ID #20)
**Changes in collaboration**	
* Collaboration with other providers*	“We collaborate with GPs and municipal health and social services to promote COVID-19 vaccination” (ID #5) “We have had good contact with municipal health and social services over the years, but our collaboration has now improved, also with GPs” (ID # 16)
* Telephone- and videoconferencing*	“In the first lockdown [of spring 2020], the collaboration was challenging, […] digital competence among employees has increased” since and “proven OAT provision adaptable to change” (ID #09) “[telephone- and videoconferencing offered] increased flexibility, […] continue to be used where appropriate” (ID #19)

## Results

Following a summary of the respondent characteristics (Participation and site characteristics section), we have structured the presentation of the results according to the two main qualitative categories*: Experience of changes in service delivery* (Experiences of changes in service delivery section) and *collaboration with other providers* (Experiences of changes in collaboration section; see also Table 1). An overview of the quantitative results from the closed-format survey items is provided in Table 2.

**Table 2:** COVID-19 related adaptions in OAT service delivery in 2021 compared to pre-pandemic levels (*n* = 23 OAT units)

	Yes		Missing/Unknown	
	*n* = *23*	%	*n* = *23*	%
*COVID-19 infection control*				
Protocol for COVID-19 cases	22	95.7	0	0
Continued use of COVID-19 safety measures	21	91.3	0	0
Availability of personal protective equipment (PPE)	21	91.3	0	0
Reduced face-to-face contact	16	69.6	0	0
Reduced outpatient treatment capacity	7	30.4	0	0
*OAT uptake*				
Stable patient demand for OAT	20	87.0	2	8.7
Faster assessment and initiation of OAT	14	60.9	1	4.3
*Medication dispensing*				
OAT home delivery: COVID-19 cases only	21	91.3	2	8.7
OAT home delivery: general	12	52.2	4	17.4
Take-home doses: longer intervals	12	52.2	2	8.7
Take-home doses: > 7 days allowed	11	47.8	1	4.3
Supervised dosing: Continued reduction	11	47.8	2	8.7
Supervised dosing: Tightened again	10	43.5	2	8.7
*Drug screening*				
Less drug screens	13	56.5	2	8.7
*Telemedicine*				
Increased use of telephone consultations	21	91.3	0	0
Increased use of video consultations	20	87.0	0	0

*OAT* Opioid agonist treatment

### Participation and site characteristics

Out of the 42 survey recipients, responses were received from *n* = 23 managers/lead physicians who were primarily female (14; 61%). They represented 23 OAT units from all 19 publicly funded health trusts, equivalent to a response rate of 100% among the health trusts. These reporting 23 OAT units are jointly responsible for the treatment of 7,789 (more than 90%) Norwegian OAT patients, with a median number of 283 patients (range: 9–1,100) per unit.

The participants’ qualitative responses to open-ended questions comprised 4,195 words in total, equivalent to 8.5 single-spaced pages of text. Participants’ individual responses ranged from 0 to 63 words in length.

### Experiences of changes in service delivery

In 2021, most OAT units (91.3%) still practiced revised operating procedures for COVID-19 infection control as established in the beginning of the pandemic. The most common adaption was the use of special routines for suspected or confirmed COVID-19 cases (95.7%).

#### Medication dispensing

Whereas all responding sites (91.3%) continued to offer OAT home delivery for patients with active COVID-19 infection, only one in every two sites (52.2%) provided home delivery to non-COVID-19 cases. The OAT units were split on the issues take-home doses and supervised dosing: Just over half maintained extended take-home intervals for OAT medications (52.2%), of which only eleven (47.8%) continued to provide take-home doses for more than 7 days. Eleven units maintained the pandemic-related reductions in supervised dosing (47.8%), and 10 units (43.5%) tightened supervised dosing practices again, with information from two sites missing. Survey respondents expressed both positive and negative experiences related to these changes*.*

Respondents from two units reported anecdotal evidence of a greater extent of diversion (i.e., sharing and selling of OAT medications) due to more liberal dispensing. However, many units reported that patients handled the COVID-19-related adaptions well:

*“We have moved form a standardized approach to more individual treatment in each case. Patients express more satisfaction with OAT in every way. Treatment availability is improved with telemedicine and more outreach work. We have increased our availability across all channels. lt is easier for our patients to contact us. We have seen surprisingly good effect of the reduction in supervised dosing and drug screens (ID #16)”*

*“Positive collaboration with most patients in terms of infection control. Patients spend less time collecting the medication, which improve their quality of life. Home delivery of the medication has allowed us to get a better sense of the patients’ living situation and needs (ID #1)”*

Several units maintained more liberal dispensing routines if individual patient assessments allowed. For instance, one unit that had entirely waived the requirement for supervised dosing in the first year of the pandemic reported that they revised clinical practice in the second year to tighten safety measures, with only long-term stable patients being exempt from supervised dosing. A respondent from yet another unit commented that *“it [was] easier to relax safety measures than to tighten these again”* (ID #5).

#### The use of depot buprenorphine

Regarding medication choice, respondents from two units (ID #04, #09) specifically mentioned patients switching buprenorphine medications, from daily oral formulations to the use of depot injections. They reported increased patient demand for depot injections, which were now being used in almost one third of patients of their unit (ID #04): “*Many patients express positive experiences and satisfaction with [depot buprenorphine], where for some the pandemic has been decisive for this medication choice.”*

#### Drug screening

Compared to pre-COVID-19 levels, most OAT units (56.5%) continued to request less drug screens from patients. A respondent confirmed that “*urine drug screening has been significantly reduced” (ID #23),* and some providers mentioned having *“largely switched to saliva samples” (ID #01, ID #10).* As one respondent explained, *“during the entire COVID-19 period, individual assessments have been made [for] urine drug screening, supervised dosing, and pick-up intervals for medications, with the aim to strike a balance between treatment safety and risk of infection”* (ID #08). The use of urine drug screening as a safety measure was thus limited in several units to individual assessments and based on patient need. For instance, this could be where patients needed to document abstinence for their driver’s license or for child welfare services or wished to obtain a more flexible OAT dispensing schedule or where providers perceived clinical need due to comorbidity (e.g., attention deficit hyperactivity disorder or chronic obstructive pulmonary disease) and co-prescribing (benzodiazepines) (IDs #10, #11, #16). Patients’ substance use was identified as key factor necessitating a reintroduction of safety measures: *“Recently, urine drug screening has been resumed for some patients where there has been a need to assess their drug use.” (ID #11).*

#### Telemedicine

The respondents reported increased use of phone (91.3%) and video consultations (87.0%) in direct contact with patients. However, some pointed to the challenges posed by patients’ *“lack of digital equipment”* or *“lack of phone and video access”* (ID #01, ID #08), requiring face-to-face outdoor meetings instead (ID #16). Several providers remained critical of telemedicine, explaining that *“phone and video cannot compensate for face-to-face contact”* (ID #19) as one would lose *“the interaction of joint meetings with the patient” (ID #05)* as well as *“essential observations in treatment, such as a patient’s smell, skin color, tremor, *etc*.”* (ID #01). Another respondent likened the use of telemedicine to some patients *“hav[ing] become more isolated as there are fewer face-to-face meetings” (ID #20).* One respondent specifically mentioned the most vulnerable patients’ need for face-to-face contact (ID #8).

### Experiences of changes in collaboration

#### Collaboration with other providers

In general, the collaboration between OAT units in the hospital trusts and municipal health and social services were perceived as positive, both before and during the pandemic. Several OAT units underlined the importance of collaboration during COVID-19, especially regarding infection control and vaccination. As one respondent wrote: *“We collaborate with GPs and municipal health and social services to promote COVID-19 vaccination* (ID #5)”. Another survey participant commented:* “We have had good contact with municipal health and social services over the years, but our collaboration has now improved, also with GPs (…) (ID # 16)”.*

However, one respondent also noted that collaboration with municipal health and social services was negatively impacted by social distancing measures with staff working from home (ID #7).

#### Telephone- and videoconferencing

Telephone- or videoconferencing was frequently used in collaboration with municipal partners, GPs, housing managers, and substance use consultants. One respondent highlighted that telecommunication with colleagues reduced travel between sites, saved time, and facilitated collaboration and decision-making. A respondent explained: “*In the first lockdown [of spring 2020], the collaboration was challenging”,* adding that *“digital competence among employees has increased”* since and *“proven OAT provision adaptable to change”* (ID #09). Another respondent noted the *“increased flexibility”* with telephone- and videoconferencing, which would *“continue to be used where appropriate”* (ID #19).

## Discussion

Our findings indicate that Norwegian OAT providers had mixed experiences with the continued flexible delivery of OAT treatment in the second year of the COVID-19 pandemic, both in interaction with patients and other providers. Nearly all Norwegian OAT units continued to experience the strain of restrictions on face-to-face treatment provision, in line with other reports and reviews from the beginning of the pandemic [16, 25]. In response, most OAT sites maintained three central COVID-19 adaptations, namely special protocols for COVID-19 cases, the use of telemedicine, and extended intervals for OAT take-home doses. According to the providers in our study, patients, by and large, handled these adaptations well.

Indeed, increased treatment flexibility including unsupervised dosing is generally welcomed by patients, for whom it reduces the burden of treatment and access barriers [7, 26, 27]. However, fear that more take-home doses and fewer monitored intakes may increase the risk of diversion and increase overdose mortality is a common concern among clinicians and policymakers [3, 8, 28–30]. In the UK, take-home provision of OAT during the first COVID-19 lockdown was associated with an increase of 64% in methadone mortality not among OAT patients themselves, but in the wider community [31]. In the present study, only few sites reported that the opioid agonist medicines had been shared or sold by patients. Nonetheless, several sites scaled back on the deregulation of OAT provision by reintroducing shorter take-home intervals, more supervised dosing, and less liberal dispensing. By the end of the second pandemic year, less than half of OAT units allowed for take-home doses beyond seven days.

For reference, Norwegian patients picked up their OAT medication on average 3 times a week in 2021, of which 2.9 events were supervised. This is only a slight reduction from the year 2019, i.e., pre-pandemic clinical practice, when patients were required to attend their OAT on average 3.6 times per week, of which on average 3.5 dosing events were supervised [21]. (NB: For example, a 7-day take-home dose implies once weekly dispensing. Since OAT dose dispensing does not always involve supervised dosing, Norwegian OAT units report the frequency of both events as separate figures as part of routine monitoring in the national annual OAT status reports [21]. Frequencies are not reported separately for buprenorphine vs. methadone).

In our study, patients’ substance use was mentioned as reason for re-introducing safety measures in the present study. However, the variation in practices between OAT units may also reflect more general differences in treatment orientation, with some units being more focused on safety measures than others. For instance, according to the Norwegian annual status report, OAT units requesting regular urine drug screens ranged from 21% (Mid-Norway region) to 45% (West region) of patients [21].

Finding the right balance between access to life-saving OAT medications and treatment safety (e.g., diversion) is a well-known dilemma [28]. During the pandemic, clinicians also had to balance treatment access and the risk of diversion of OAT medications with patients’ risk of COVID-19 infection when collecting OAT medications. Stricter dosing regimens for less stable patients are common and may benefit treatment engagement and adherence [2, 6, 30]. Supervised dosing comes at a higher service cost for providers but may provide structure and social support for some patients [7, 32]. To illustrate this point, Harris et al. [32] reported that some patients viewed daily attendance of the OAT unit as important for starting their day as well as for their long-term stability. In a systematic review [2], supervised dosing had no effect on treatment retention.

In our study, the (partial) re-tightening among OAT units was offset by significant innovation that occurred in the Norway OAT system during the COVID-19 pandemic, most notably with the scaling up of telemedicine and depot buprenorphine. In contrast to most countries in Europe [28], buprenorphine is the most prescribed medication in OAT in Norway. Depot buprenorphine was introduced in the Norwegian OAT program in 2019, and 2% of the patients were prescribed depot buprenorphine at the end of 2019. However, its use multiplied during the COVID-19 pandemic, as 15% of the Norwegian OAT patients were prescribed depot buprenorphine by the end of 2021 [21]. This trend is consistent with increased use of depot buprenorphine in Australia [9] where patients in a randomized trial also reported improved treatment satisfaction compared to those receiving sublingual buprenorphine [33]. One advantage with this formulation is that patients need to attend the clinic less often. However, further research is needed to identify which patients benefit most from extend-release formulations.

To compensate for reduced face-to-face meetings with patients, OAT units in Norway introduced video and telephone consultations at the start of the pandemic [15]. These became an integral part of treatment in the second year of the pandemic, even as in-person consultations resumed. The use of real-time telemedicine has accelerated during the COVID-19 pandemic for several health conditions, including treatment for substance use disorders [34, 35]. Telemedicine may improve access to OUD treatment [12–14, 36]. A recent US cohort study shows that receipt of OUD-related telemedicine service during the COVID-19 pandemic was associated with improved treatment retention and lower odds of overdose compared to pre-pandemic levels [37]. The use of telephone and videoconferencing also improved collaboration with other providers, and reduced travel time. While OAT providers in our study generally perceived the use of video and telephone communications as positive in collaboration with other providers, some noted that the (exclusive) use of telemedicine made clinical judgement more difficult, as significant information (e.g., visual or non-verbal cues, smell) about patients’ well-being was not accessible. In the literature, both clinicians and patients report that some patients benefit from more structure and regular meetings with therapists [7, 8]. Clinicians are more comfortable using telemedicine for clinical stable compared to unstable patients [38, 39].

### Implications for policy and clinical practice

Our findings suggest that telemedicine can be useful as adjunct to face-to-face treatment and provide greater flexibility for patients, who according to Lockard et al. [40] should be offered the choice between modalities, where possible. Telemedicine may be particularly suitable for rural regions. In line with our findings, vulnerable populations need attention [34], as lack of digital literacy, lack of equipment or lack of privacy may be a barrier for some patients [38, 41]. Thus, there is an urgent need to study what treatment modalities (remote vs. in-person) work for whom.

In recent years, both patients and providers have voiced the need for stronger emphasis on user involvement and patient choice. The Norwegian OAT guideline from 2010 was revised in 2022 [19]. According to the revised guideline, Norwegian OAT patients should receive holistic and well-coordinated treatment, with a high degree of user involvement. COVID-19 has in some ways served as a trial period for the revised guidelines, with preliminary evidence that more individualized OAT provision can be maintained. Further evaluation of the revised guideline is forthcoming.

### Strengths and limitations

Our study has at least four limitations. Firstly, as with any self-reported study, recall bias may limit the internal validity of our findings. Secondly, the free-text option in the questionnaire meant that respondents may not have entered the same level of detail, and our analysis of the qualitative data was therefore limited to basic Iterative Categorization. Thirdly, due to the sample size and limited statistical power, no group comparisons or inferential statistics were performed. Finally, since this study focused on provider experiences of OAT service delivery, no data on OAT patient perspectives or health outcomes were collected. A Norwegian study found high awareness of COVID-19 symptoms and available services among OAT patients [42], but there remains uncertainty as to how changes to OAT delivery during the pandemic have impacted health outcomes. Triangulation of patient and provider perspectives will may further improve our knowledge of factors impacting quality of care in OAT delivery in the post-pandemic era.

Nevertheless, this cross-sectional study included OAT units from all 19 hospital trusts, covering more than 90% of patients receiving OAT in Norway. The results are considered representative of the Norwegian OAT program. While it is unclear how generalizable our findings are to OAT settings in other countries, it is important to monitor providers’ ability to adopt and adapt to clinical innovation and share lessons learnt internationally as part of this evolving process.

## Conclusions

Overall, the Norwegian OAT system proved resilient in the second year of the COVID-19 pandemic, as its healthcare workforce embraced innovation in technology (telemedicine) and drug development (depot buprenorphine). According to our nationally representative sample of OAT providers, most patients handled increased take-home doses of methadone and buprenorphine well. Our findings suggest that telemedicine can be useful as adjunct to face-to-face treatment and provide greater flexibility for patients.

## Data Availability

The dataset generated and analysed during the current study is not publicly available to protect the privacy of participants, but it is available from the corresponding author on reasonable request.

## Abbreviations

OAT: Opioid agonist treatment
COVID-19: Corona Virus Disease 2019
